# Dynamic biospeckle analysis, a new tool for the fast screening of plant nematicide selectivity

**DOI:** 10.1186/s13007-019-0523-8

**Published:** 2019-12-18

**Authors:** Felicity E. O’Callaghan, Roy Neilson, Stuart A. MacFarlane, Lionel X. Dupuy

**Affiliations:** The James Hutton Institute, Invergowrie, Dundee, D2 5DA Scotland, UK

**Keywords:** Dynamic speckle, Isothiocyanate, Nematodes, Selective plane illumination microscopy (SPIM)

## Abstract

**Background:**

Plant feeding, free-living nematodes cause extensive damage to plant roots by direct feeding and, in the case of some trichodorid and longidorid species, through the transmission of viruses. Developing more environmentally friendly, target-specific nematicides is currently impeded by slow and laborious methods of toxicity testing. Here, we developed a bioactivity assay based on the dynamics of light ‘speckle’ generated by living cells and we demonstrate its application by assessing chemicals’ toxicity to different nematode trophic groups.

**Results:**

Free-living nematode populations extracted from soil were exposed to methanol and phenyl isothiocyanate (PEITC). Biospeckle analysis revealed differing behavioural responses as a function of nematode feeding groups. *Trichodorus* nematodes were less sensitive than were bacterial feeding nematodes or non-trichodorid plant feeding nematodes. Following 24 h of exposure to PEITC, bioactivity significantly decreased for plant and bacterial feeders but not for *Trichodorus* nematodes. Decreases in movement for plant and bacterial feeders in the presence of PEITC also led to measurable changes to the morphology of biospeckle patterns.

**Conclusions:**

Biospeckle analysis can be used to accelerate the screening of nematode bioactivity, thereby providing a fast way of testing the specificity of potential nematicidal compounds. With nematodes’ distinctive movement and activity levels being visible in the biospeckle pattern, the technique has potential to screen the behavioural responses of diverse trophic nematode communities. The method discriminates both behavioural responses, morphological traits and activity levels and hence could be used to assess the specificity of nematicidal compounds.

## Background

Nematodes are worms of microscopic size which have been highly successful in colonising a wide range of ecosystems [1]. Soil nematodes move within the soil matrix by making use of the films of water present between adjacent soil particles [2]. Soil nematodes can be categorised into different trophic groups, such as bacterial feeders, plant feeders, fungivores, predators and omnivores [3]. Plant feeding nematodes cause significant damage to crops worldwide either by directly feeding on plant roots or by transmitting viruses such as tobra- and nepoviruses [4], leading to significant economic costs through crop losses and control measures [5]. On the other hand, nematodes have a beneficial effect on soil health, both in terms of agricultural production and sustainability [6–8]. Often, there is a strong link between nematode trophic group, reflecting their feeding behaviour, and their function [9]. Bacterial feeders in particular have been associated with higher levels of mineralised nitrogen [10, 11], making nutrients available to plants by acting as decomposers [12], and dispersing and aiding colonisation by beneficial bacterial communities [13, 14]. Large populations of bacterial feeding nematodes have also been found to positively affect root growth [15].

The development of environmentally-friendly forms of nematode control has been hampered by the lack of methods able to consider interactions within the biosphere. Previous approaches to studying the effect of any chemical treatment on nematodes were based on direct measurements such as responsiveness to touch or the uptake of a stain, both of which were determined by eye, to assess viability or fecundity [16–18]. A main limitation of these techniques is that they mostly focus on a single criterion and overlook all other forms of bioactivity, which can be subjective and lead to erroneous deductions. Additionally, these types of analyses are time consuming to perform and difficult to automate. Increasingly, soil nematode identification is being superseded by molecular ID tests (for example [19–21]), while in toxicity testing, molecular techniques are now proving a highly useful tool in investigating the mode of action of pesticides [22, 23]. However, although knowledge of the genomes of in-soil biota is steadily expanding, significant gaps remain with most barcoding studies being targeted to a fraction of the biodiversity present [24], and treatment effects need to be inferred from changes to the abundance of assayed organisms when sampled over time. Furthermore, genomic approaches, while able to distinguish between different species of nematode, are unable to take into account the behaviour patterns which determine the functions of different nematodes in the ecosystem. Increasingly, calls are being made for the development of methodologies to not only focus on population composition and abundances but to also consider their levels of activity and interaction [6, 25, 26]. For soil management, identifying changes to the relative proportions of different functional or trophic groups has been proposed as a useful, less cumbersome alternative [27, 28].

Optical screening has recently undergone a transformation both in terms of throughput and capability. This is particularly evident in human drug discovery using the nematode *C. elegans* as a model organism [29]. A wide array of screening methods is now available to automatically screen *C. elegans* for behavioural responses and morphological changes [30–32]. For example, worm tracking software can be used to identify chemical-induced changes or inhibition of movement [33, 34]. Though some techniques require mutants expressing fluorescent markers for better contrast, others have no such constraints, thereby potentially enabling a wider range of species to be screened. Such label-free techniques base their analyses on measures of pixel change in a series of bright or dark field images [35–37].

Here we propose to exploit a phenomenon known as biospeckle. Biospeckle occurs when a living biological organism is illuminated by a laser. Coherent light produces interferences when interacting with living tissue [38], and the technique is known to improve contrast compared to bright field imaging without the need for labelling or staining. Biospeckle has previously been exploited for measuring movement, such as of blood, spermatozoa or motile bacteria [39–41], or assessing the extent of infection or decay in biological tissues [42–45]. More recently the technique has been applied to nematology as a tool for pharmacological tests on parasitic larvae [46] and for the detection of soil nematodes [47]. We previously demonstrated the successful distinction between live and heat-killed nematodes [47]. In this paper, we develop an approach that utilises biospeckle generated in a light sheet microscope to acquire patterns that reflect responses of nematodes to chemicals. Here, the biospeckle approach for screening is applied directly to the sampled population and not restricted to an identified subset; it is rapid and requires minimal input by the experimenter.

## Methods

### Image acquisition

**Fig. 1:**
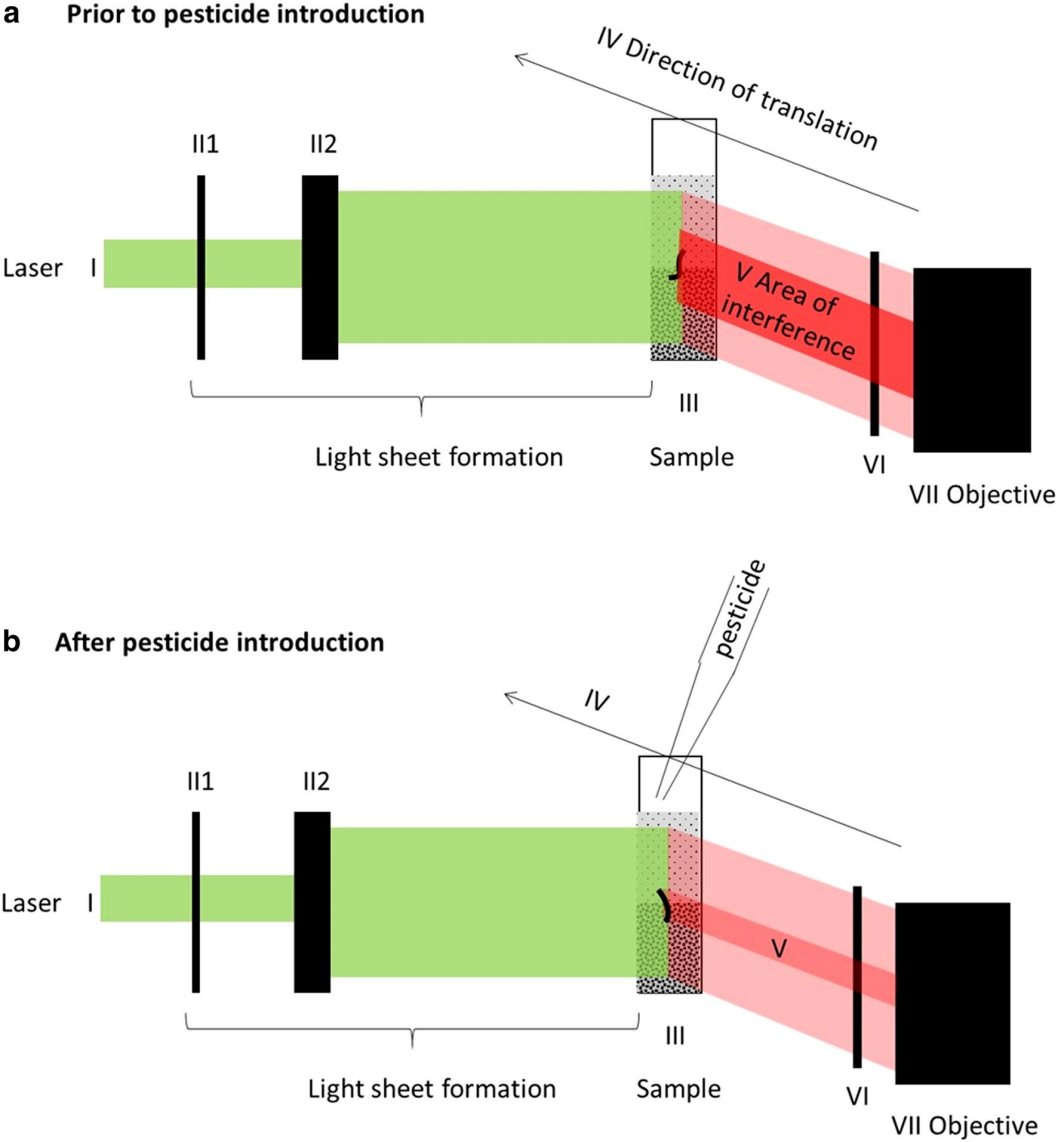
Experimental set-up and principle of bioactivity quantification through biospeckle. A light sheet was formed by passing the beam of a 1.5 mW 520 nm laser (I) through a 1 mm slit (II1) and a vertical cylindrical lens (II2). The light sheet cuts an optical section through the Ludox® TMA /water sample (III) containing the nematodes. A motorised stage (not shown) translates the sample at an axis at right angles to the laser beam. Translation proceeds in a series of steps, at each of which 64 brightfield images are taken. Images were taken perpendicularly to the light sheet, with a polarising filter (VI) set in front of the objective of the stereomicroscope (VII). Following image acquisition, images were processed (VIII) to create a map of speckle activity for each optical section. In theory, an active nematode, as schematised in **a**, produces interference within the laser light sheet which is proportional to its bioactivity. This interference is detectable as speckle, an area of bright voxels on the biospeckle map. After the introduction of a bioactivity inhibiting compound (**b**) however, the decline in nematode bioactivity leads to a decline in the interference within the light sheet. The biospeckle signal becomes fainter, and the number of voxels that are brighter than the background declines

Image data on the bioactivity of nematodes in each sample was obtained using the newly developed imaging technique BSPIM (Biospeckle Selective Plane Illumination Microscopy) [47]. For this, light sheets were formed with a green laser diode (Thorlabs, CPS520) emitting a beam of 520 nm wavelength at 4.5 mW intensity (Fig. 1 section I) which was formed into a light sheet of 1 mm thickness (II1–II2) by passing it through an adjustable slit (Thorlabs VA100/M) and a cylindrical lens (Thorlabs LJ1878L2). Images were acquired with a Leica MZ16 FA stereomicroscope fitted with a 0.5 × plan achromatic objective and a Leica DFC350FX camera. A motorised stage (STANDA 8MT167) moved the sample (III) through the light sheet (IV) at a perpendicular angle by increments of 62.5 µm and 64 brightfield frames were taken with 13× magnification at a rate of 10 frames per second at each step. Image acquisition of the light passing through the sample (V) was briefly paused for 0.6 s after each step. Noise from reflection was removed by placing a polarising filter (VI) in front of the objective (VII). Stage movement and image acquisition were implemented through software developed in C++ .

### Nematode sample preparation

Chemicals were applied to two trophic groups of nematode, plant feeders and bacterial feeders, as well as targeting a single plant feeding genus, *Trichodorus*, a pest of economic relevance through its transmission of tobraviruses [4]. Bacterial feeding and plant feeding nematodes differ in their behaviour, and this is reflected in their movement. Bacterial feeders are filter feeders, continuously drawing in bacteria with the surrounding liquid by contractions of their oesophageal bulb, then forcing the liquid out while retaining the bacteria [48]. Within liquid, their movement is characterised by high wavelength, low amplitude body movements and oscillation of the head region, typical behaviour when perceiving attractants [48]. By contrast, plant feeder movement proceeds much more slowly [49]. In the absence of plant attractants, plant feeders display sporadic random movements, which are characterised by their short wavelength, high amplitude and the absence of head oscillations [48].

**Fig. 2:**
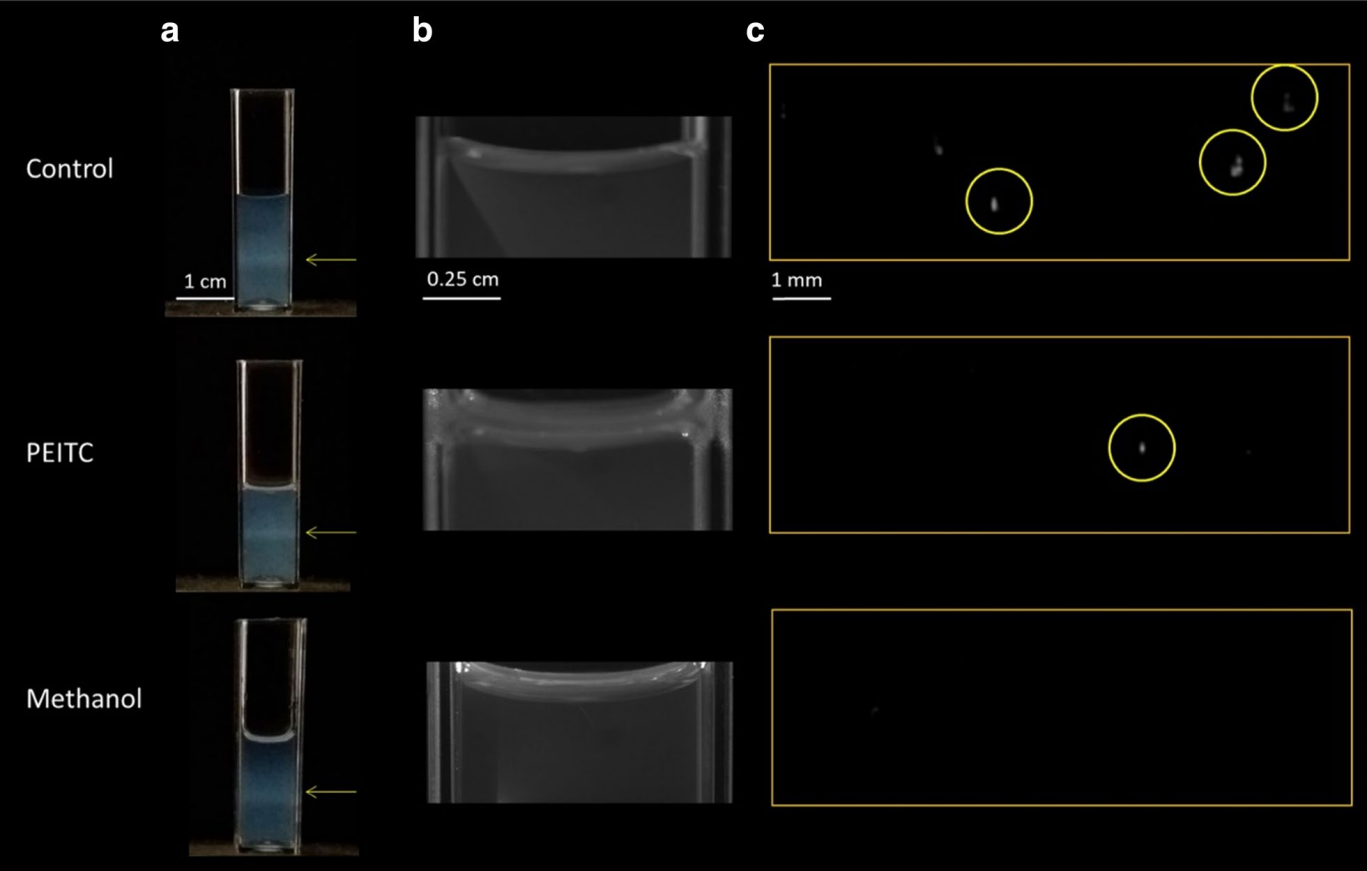
Sample preparation and object detection with 5 nematodes per cuvette. **a** Sample cuvettes containing 1 mL Ludox to 1 mL water with the Ludox® TMA/water mixing layer containing the nematodes indicated by yellow arrows. **b** Water–air boundary with PEITC, methanol or water added for controls; PEITC as an oil formed a film on top of the water column, while methanol dissolved. **c** Examples of the numbers of bacterial feeders detected out of 5 with a high brightness detection threshold. A high threshold was set to ensure that speckle from sources other than the nematodes is excluded

All samples were taken from Scottish cultivated brown soils. Nematodes were extracted from soil by sieving and by a modified Baermann funnel method [50]. For this technique, soil sieved to a particle size of < 1 mm was placed on a fine mesh of around 250 µm and placed on top of a column of tap water contained within a funnel. Nematodes migrate out of the moistened soil through the mesh to the bottom the funnel where they are then collected. The method ensures that only live and motile nematodes are extracted. *Trichodorus* species were identified to genus level, while plant (excluding trichodorids i.e. all *Trichodorus* and *Paratrichodorus* individuals) and bacterial feeders were mixed, multi-genus groups sampled from Scottish agricultural soils. To prepare nematode samples for scanning, 1 mL Ludox® TMA (Sigma 4420859) was added to each cuvette. Nematodes were picked from a watch glass by allowing them to attach themselves to a micro-pin, and then immediately transferring the micro-pin to the sample, where the nematodes were carefully floated onto the surface of the colloid. 1 mL of water was then added which resulted in nematodes being suspended in the Ludox® TMA/water mixing layer around the midpoint of the cuvette (Fig. 2a). Each sample for biospeckle analysis contained 5 nematodes and was replicated 5 times for each treatment (except PEITC tests on *Trichodorus* nematodes which had 6 replicates to ensure similar detection rates with nematodes in these samples tending to be smaller in size). Pesticide testing using BSPIM was carried out in polymethacrylate cuvettes (Sigma, C0793). In addition to the samples used in the scanning experiments, 5 replicate samples of 5 nematodes of each target feeding type were kept in Ludox® TMA/water for 1 week after which the presence of spontaneous movement was assessed by light microscopy.

### Introduction of chemicals

Two toxins were tested. Phenethyl isothiocyanate (PEITC), a naturally occurring nematicide [51] which is being considered as a replacement for synthetic controls [52], and methanol, which is occasionally applied in soil remediation [53] and is known to affect nematode feeding activity [22, 54]. Chemicals were added to the samples by pipetting them onto the water surface without disturbing the Ludox® TMA/water column. There is considerable variation in the concentration of PEITC at which suppressive effects on plant feeding nematodes have been reported (several orders of magnitude), depending on the substrate and the use of solvents [51, 55]. As solvents were not used in this study, a dosage at the upper end of this range was chosen by adding 10 µL of PEITC (Sigma, 99%, 253,731). Methanol (Fisher, M/4056/17) was added at 0.5 mL, which is the dose predicted to result in 100% lethality of *C. elegans* [54]. As an oil, PEITC formed an emulsion visible as a thin film from which compounds were allowed to diffuse, while methanol dissolved (Fig. 2b). The first BSPIM scan was completed prior to the introduction of the chemical treatments, after which further scans were carried out 2 h and 24 h post-treatment. For controls, 0.5 mL of water were added in the same manner as the chemicals.

### Biospeckle analysis

FIJI ImageJ software was used for image analysis, using the protocol described previously [47] based on the generalized differences method [56]. The 64 brightfield images taken at each step were transformed into a single map of biospeckle activity (Fig. 1). This was repeated for each step before the resulting maps were stacked together to give a 3D volume of biospeckle activity and the image cropped to a region of interest comprising the suspended nematodes. The cropped region was background subtracted with a 2 mm radius and filtered with a median filter and then a 3D Gaussian blur. ImageJ 3D object counter plugin [57] was used to identify regions of biological activity. This was done by setting a high brightness threshold and a minimum object size of 20 to reliably distinguish nematodes from background noise.

Bioactivity per sample was characterised by the Total Biospeckle Intensity (total biospeckle intensity of detected objects in a scan, Table 1) which was determined from the intensities of the detected objects and equals the sum of mean pixel intensity of all the objects detected within a sample. For chemical testing, paired t-tests were carried out to compare Total Biospeckle Intensity for each of the 5 replicates and at 0, 2 and 24 h after treatment. The effect of chemical treatment on each individual nematode may manifest itself in different ways. While a chemical compound may have an effect on nematode motility, internal processes, such as organ and cell functions may be affected differently. We therefore included two additional descriptors of the biospeckle activity of individual nematodes. The first descriptor, biospeckle intensity (mean intensity of the voxels of a detected object, Table 1), was used to assess internal changes in bioactivity, while biospeckle volume (number of voxels of a detected object, Table 1) was used to measure nematode morphology and movement. Biospeckle intensity was determined as the mean grey values of the voxels of the detected object. Biospeckle volume was calculated as the number of object voxels and relates to the overall space explored by a nematode during each BSPIM scan.

**Table 1:** Image analysis parameters and their mathematical description

Term	Definition	Formula
Speckle	Spike in light intensity captured by the camera and created by light interference in a heterogeneous medium	
Biospeckle	The dynamic speckle generated by living organisms. Speckle can be created by internal, cellular processes or body movement. Biospeckle data are in the form of 3D volume data, namely a set of voxels *Ω* with width (*w*), height (*h*) and depth (*d*), resulting from the BSPIM system	$\Omega ={\left\{{X}_{j}\right\}}_{j<w\times h\times d}$
BSPIM	Biospeckle Selective Plane Illumination Microscopy; an imaging method that quantifies the activity of biological organisms within 3D volumes through the analysis of dynamic speckle	
Detected biospeckle object	A subset of the volume image data generated by BSPIM. The subset is a connected region detected above background noise. The *i*th detected object is a subset *Ω*_*i*_	*Ω*_*i*_
Biospeckle volume	Number of voxels in the *i*th object	${N}_{i}=n\left({\Omega }_{i}\right)$
Biospeckle intensity	Mean of grey values *X*_*j*_ of the *i*th detected biospeckle object. Specific to each nematode	${I}_{i}=\frac{1}{{N}_{i}}{\sum }_{{X}_{j}\in {\Omega }_{i}}{X}_{j}$
Total Biospeckle Intensity	Sum of mean object intensities. Gives the total biospeckle intensity for the whole sample	$\mu =\sum {I}_{i}$
Biospeckle pattern	Distribution (position and intensity of pixels) of a detected biospeckle object *Ω*_*i*_. Patterns can be described by several parameters such as intensity, area, volume and circularity	
Biospeckle area	A set of connected pixels in the 2D plane maximizing the surface area of the detected biospeckle object	$S={A}_{k}$
Circularity of biospeckle area	Circularity of fitted convex hull of surface area *S* and perimeter *P*	${C}_{A}=4\pi S/{P}^{2}$

We also studied how to discriminate nematode feeding types and their response to chemical compounds using shape descriptors. In order to achieve this, four nematodes were selected from the control samples of each trophic group. A further 4 nematodes imaged after 24 h exposure to PEITC were selected from PEITC samples. The 3D viewer plugin [58] was used to obtain 2D projections from which the nematodes' biospeckle areas were measured in the XY plane (facing the objective) and YZ plane perpendicular to the laser beam. A convex hull was fitted to each selected biospeckle area. Shape descriptors were then derived from the 2-dimensional selected area. Circularity of each biospeckle area was estimated from the convex hull perimeter, measured as Circularity = 4π * hull surface area/perimeter^2^.

## Results

### Automatic detection of nematode response (Figs. 2, 3)

**Fig. 3:**
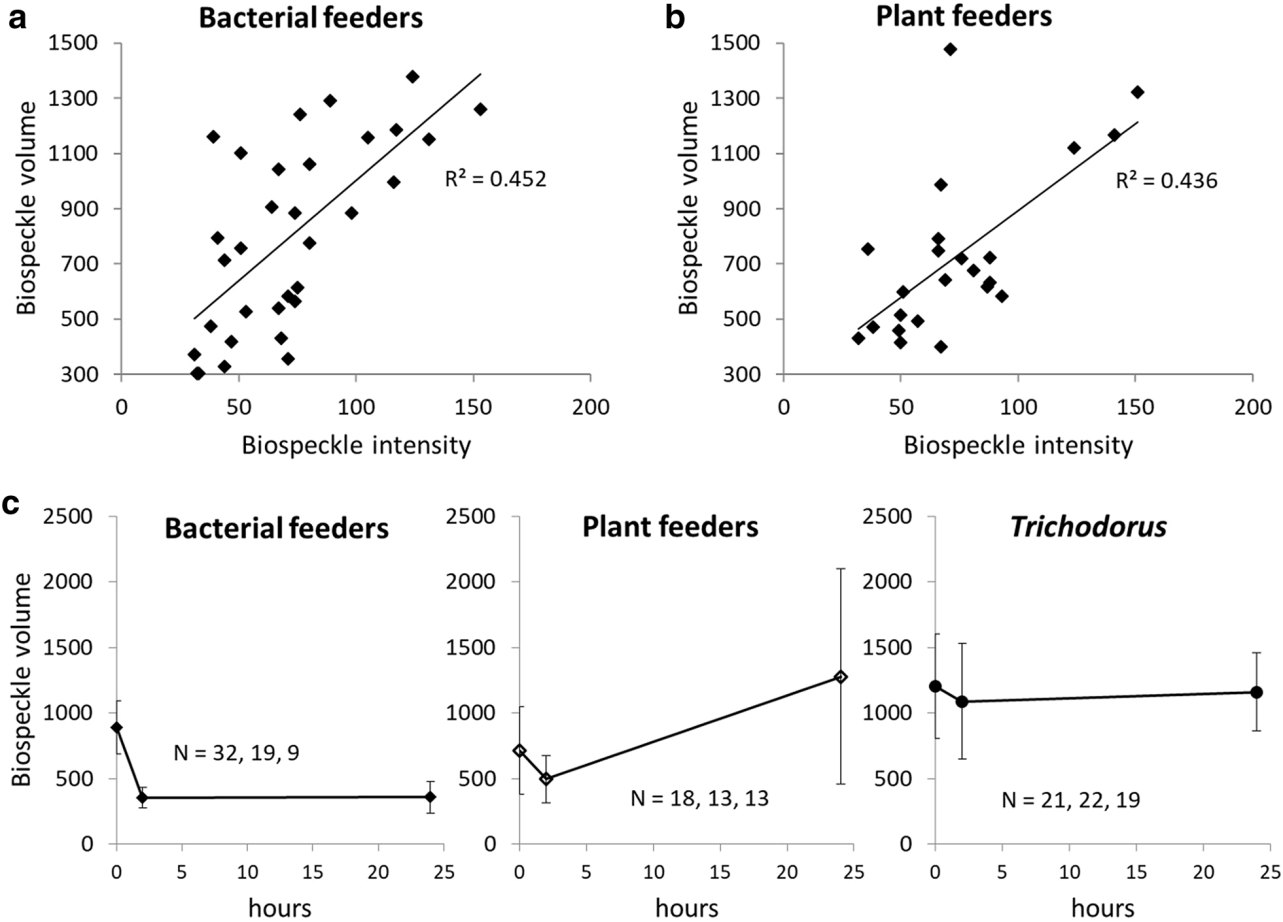
Biospeckle intensity and biospeckle volume. **a**, **b** Distribution of the biospeckle intensity of detected objects, measured in grey scale, and its correlation with biospeckle volume. Data points represent individual nematodes which were imaged at 5 individuals per cuvette. Within the range of 300 to 1500 in biospeckle volume, N = 34 for bacterial feeders and N = 24 for plant feeders. R^2^ values for linear regressions are 0.45 for bacterial feeders and 0.44 for plant feeders. **c** Changes to biospeckle volume over time, in the absence of chemical treatment. Data from 5 replicate samples for each nematode type, with 5 nematodes contained in each sample. For bacterial feeders biospeckle volume decreased after insertion, while plant feeders showed large variation and biospeckle volume of *Trichodorus* nematodes remained stable. Biospeckle volumes are shown as the average of all detected objects; N values are given for each of the 3 sampling times and error bars represent the standard error

Using the BSPIM technique, it was possible to map the biospeckle activity of assemblages of nematode within transparent 3D volumes. We show that BSPIM is suitable as a nematode detection tool, but also to track changes in nematode bioactivity in response to the application of chemicals. To this end, a region of interest comprising the Ludox® TMA/water mixing layer in which the nematodes were suspended was specified for each sample (Fig. 2c). Biospeckle maps visualising the activity of each assemblage of 5 nematodes were obtained (1) after nematode insertion, before treatment application, (2) at 2 h and (3) at 24 h post-treatment. From each map, the Total Biospeckle Intensity within each sample was measured for each time point. Declines in movement or internal biological activity, whether as an effect of time or chemical treatment, lead to a decline in biospeckle and even to non-detection at above noise thresholds (see Fig. 2c for an example). Members of a feeding group exhibit characteristic movements and activity levels that help to differentiate them from other feeding groups. Also, within a feeding group, age or genetic differences may induce other forms of activity patterns that cannot be studied with a unique descriptor. Hence, descriptors that are specific to each individual nematode must be used to improve the analysis of nematode bioactivity. In the next step, we analysed variations in the volumes of detected objects (biospeckle volumes, Table 1) and the intensity of the bioactivity of detected objects (biospeckle intensity, Table 1). Analyses were first carried out within a single scan, and then analysed for variability over time. Biospeckle volume relates to motility while biospeckle intensity indicates the internal biological processes of nematodes.

We observed correlations between the biospeckle volume of a detected object and other nematode attributes linked to biological activity, body size and motility. First, there is an association between biospeckle volume and the biospeckle areas in successive frames. A mature nematode, because of its larger size, will be detected as a larger object (biospeckle volume) and this can be confounded with a smaller nematode moving across larger distance during the scan. However, nematode body size in a sample does not significantly change during the experiment, though any fluctuations in biospeckle volume reflect changes in internal bioactivity or motility. Our results show the biospeckle volumes of detected objects to be correlated with their biospeckle intensity for both bacterial and plant feeders (Fig. 3a, b respectively). Pearson’s correlations were highly significant for both bacterial and plant feeders (n = 96 and n = 57 respectively and p = 0.000 for both). This correlation is due to two potential effects that could not be separated during this study. First, larger nematodes may have higher levels of internal bioactivity, and second, movement faster than the acquisition rate causes increases in biospeckle intensity. Over time and in the absence of chemical treatment, biospeckle volume for detected nematodes only remained constant in the case of *Trichodorus* nematodes (Fig. 3c). For bacterial feeders, biospeckle volume reflecting nematode activity declined over time, while measurements for plant feeders showed a high level of variation.

### Nematode trophic groups are affected differently by chemical compounds (Fig. 4)

**Fig. 4:**
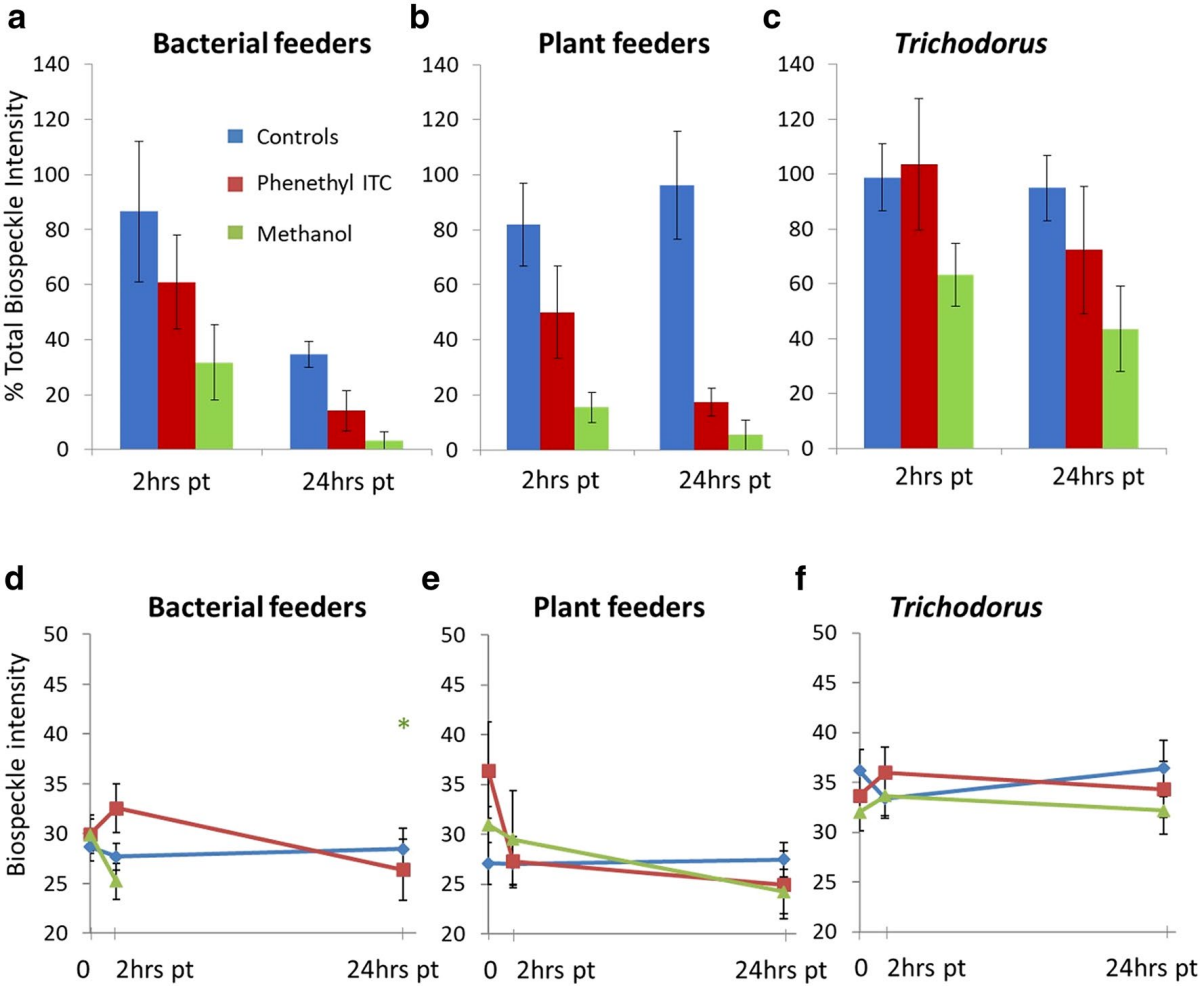
Effects of PEITC and methanol on biospeckle intensity (see Table 1 for definition of parameters). **a**–**c** Changes in the Total Biospeckle Intensity of bacterial feeder, plant feeder and *Trichodorus* samples 2 and 24 h after treatment, expressed as the percentage of Total Biospeckle Intensity measured before treatment. Error bars represent the standard error for N = 5 (N = 6 for *Trichodorus* in PEITC). **d**–**f** Biospeckle intensities for the different feeding groups detected above a threshold of 20. Measurements at 0 h are prior to the application of chemicals, while 2 and 24 h are post-treatment measurements. Biospeckle intensities are shown as the average of all detected objects and error bars represent the standard error. The asterisk denotes the only observation 24 h after the application of methanol which was not replicated

Both PEITC and methanol had measurable effects on nematode bioactivity when compared with controls. Here, bioactivity was measured as the Total Biospeckle Intensity (Table 1), a global indicator determined as total object intensity for each sample of 5 nematodes. Total Biospeckle Intensity varied considerably between the different feeding groups tested (Fig. 4), regardless of the presence or absence of the toxic compounds. For bacterial feeders (Fig. 4a), Total Biospeckle Intensity in the absence of chemical treatment decreased over the 2-day testing period, with levels significantly different after 24 h (p = 0.283 after 2 h and p = 0.013 after 24 h). The application of PEITC lead to a decline in the Total Biospeckle Intensity of bacterial feeders which was significant after 24 h (p = 0.075 and 0.007 after 2 and 24 h respectively). The introduction of methanol was immediately followed by a significant decline in Total Biospeckle Intensity (p = 0.026 and p = 0.003 after 2 and 24 h, respectively). For plant feeders (Fig. 4b), Total Biospeckle Intensity did not vary significantly for controls (p = 0.255 after 2 h and p = 0.413 after 24 h). The application of PEITC on the other hand was followed by a significant decrease in the Total Biospeckle Intensity both 2 and 24 h after application (p = 0.038 and p = 0.003). Similarly, the application of methanol was followed by a significant decline after 2 and 24 h (both p < 0.001). *Trichodorus* nematodes (Fig. 4c) were the least affected by either PEITC or methanol. While no significant difference in the Total Biospeckle Intensity over time was noticeable for controls (p = 0.784 and 0.453 after 2 and 24 h respectively), the application of PEITC also showed no significant effect after 2 h or 24 h (p = 0.355 and p = 0.072). No significant change was apparent 2 h after the application of methanol (p = 0.179), but a significant decline in the Total Biospeckle Intensity had occurred after 24 h (p = 0.035).

Biospeckle intensity is specific to each nematode (unlike Total Biospeckle Intensity, which considers the whole sample). Results showed that biospeckle intensity did not vary significantly for control samples (Fig. 4d–f). Exposure to PEITC and methanol was followed by downward trends in biospeckle intensity for bacterial feeders at 24 h (Fig. 4d) and plant feeders at 2 and 24 h (Fig. 4e), though not for *Trichodorus* nematodes (Fig. 4f). For nematodes recovered after 1 week in the Ludox® TMA/water mixing layer, spontaneous movement was observed in 15 out of 17 bacterial feeders, 11 out of 20 plant feeders, and 6 out of 23 *Trichodorus* nematodes*.* This confirmed *Trichodorus* nematodes to have the lowest motility even in the absence of toxins while bacterial feeders were the most motile, albeit displaying greater variability in velocity and distance travelled.

### Analysis of target specificity

**Fig. 5:**
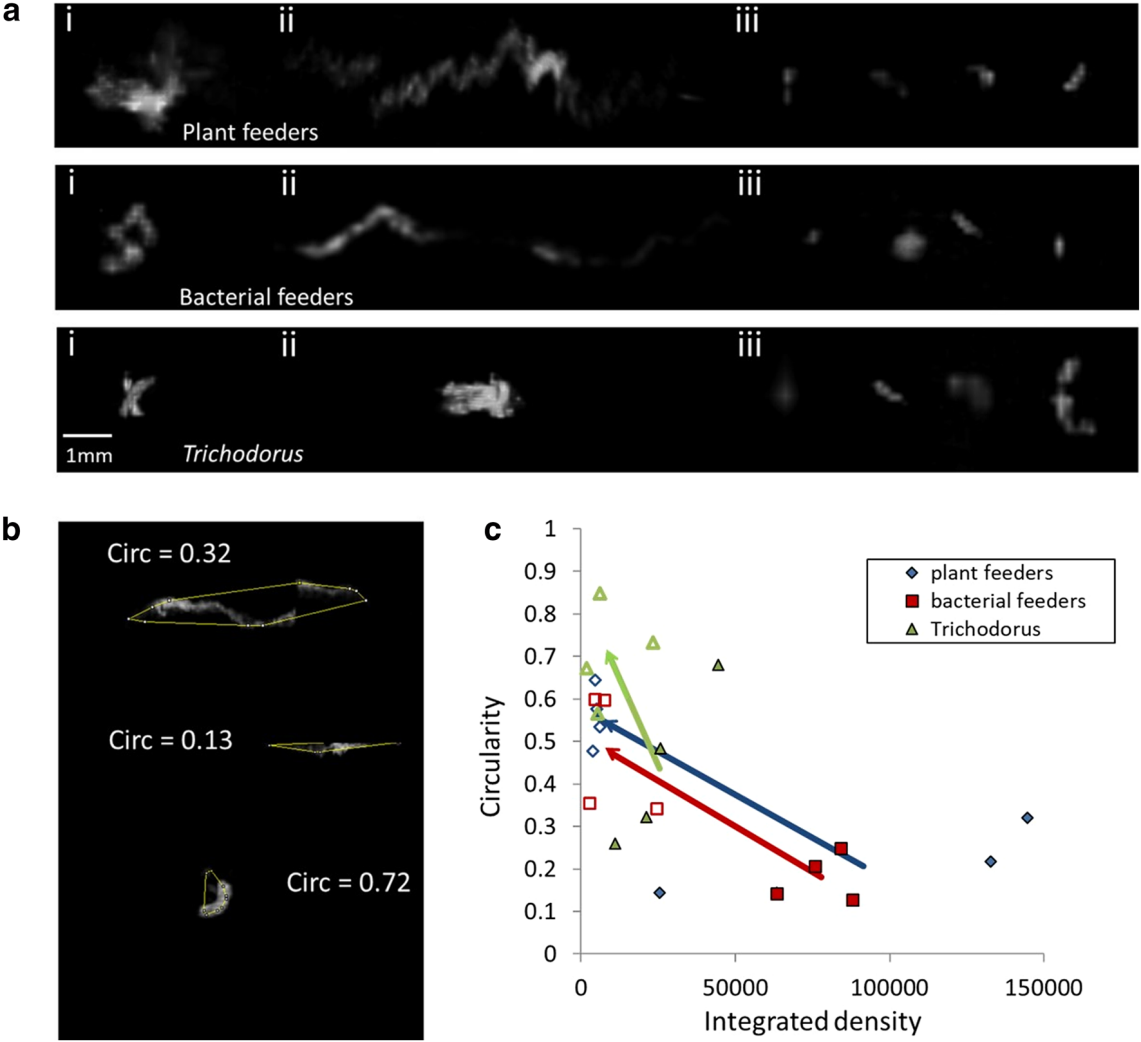
Changes to biospeckle object shape after PEITC treatment. **a** Biospeckle objects rendered in 3D from stacks of optical sections. (i) and (ii) show examples typical of object shape pre-treatment with (i) showing the x–y plane and (ii) showing the x–z plane. (iii) shows objects detected 24 h after PEITC treatment. **b** Examples of convex hull shape applied to areas of biospeckle activity rendered in 3D. Increased curvature of the biospeckle area increases the circularity (Circ) of the fitted convex hull shape. A circularity of 1 describes a perfect circle. **c** Effect of PEITC on the circularity and the integrated density of nematode biospeckle shapes. The minimum circularities of 8 nematodes (4 showing clear activity in the absence of PEITC, and 4 with clearly definable biospeckle areas 24 h after the addition of PEITC) measured in the XY and YZ plane are given. Blue markers represent non-trichodorid plant feeders, while red and green markers represent bacterial feeders and *Trichodorus* nematodes respectively. Arrows indicate the difference in mean circularity and integrated density measured for treated as opposed to untreated nematodes. In the absence of chemical treatment (filled markers), circularity for bacterial and non-trichodorid plant feeders was lower than after 24 h in PEITC (open markers). On average, *Trichodorus* nematodes had greater circularity than either bacterial feeders or non-trichodorid plant feeders in the absence of treatment and did not show any significant increase in circularity once PEITC was applied

Although biospeckle volume and biospeckle intensity varied between the different functional groups studied here, these alone are not sufficient to accurately discriminate the feeding groups. Detailed visual analysis of the data revealed numerous other features could be extracted for a discriminant analysis. In particular, there was strong morphological variation in the shapes of the areas of the detected biospeckle objects. These shapes also vary considerably with time and in response to chemical compounds, and this could easily be quantified by a shape analysis. As is illustrated in Fig. 5a, nematodes moving in 3-dimensional space give rise to low density, drawn-out biospeckle patterns. Stationary areas of high biospeckle intensity on the other hand give rise to compacted shapes. Taken together, this implies that inactive nematodes produce more circular areas of biospeckle than active, motile ones. These changes in biospeckle can be captured by different descriptors. Both biospeckle volume (Fig. 3c) and Total Biospeckle Intensity differed between the different groups of nematodes (Fig. 4a–c). As seen in the absence of chemical treatment, biospeckle volume decreased over time for bacterial feeders, was highly variable for plant feeders and remained constant only for *Trichodorus* nematodes (Fig. 3c). By contrast the biospeckle intensity of a living nematode remains relatively constant (Fig. 4d). The sharp early decline in biospeckle volume for bacterial feeders appeared greater than the decline in bioactivity of bacterial feeder controls seen previously (Fig. 4a). The findings confirmed observations made by eye in which, in the absence of toxins, plant and bacterial feeders were much more motile than *Trichodorus* nematodes (Fig. 5ai and ii). This changed after chemical treatment. Following treatment with PEITC or methanol, there was a noticeable decrease in activity for both plant feeders and bacterial feeders (as seen in the examples of nematodes treated with PEITC in Fig. 5aiii), though less so for *Trichodorus* nematodes, which moved little throughout the experiment.

We have tested descriptors such as the shape area, perimeter, integrated density and skew (data not shown). Circularity was the best single parameter at discriminating between levels of nematode motility. By using circularity as a descriptor of shape (Fig. 5b), we examined the differences in biospeckle patterns between the different trophic groups as well as the effect of chemical treatment. While plant and bacterial feeders displayed extended biospeckle patterns in 3D space, this was never observed for *Trichodorus* nematodes. Moving plant and bacterial feeders had biospeckle areas that tended to exhibit lower circularity than those of *Trichodorus* nematodes (Fig. 5c, filled markers). However, 24 h after the addition of PEITC (Fig. 5c, open markers), circularity increased in bacterial feeders and non-trichodorid plant feeders (p = 0.009 and p = 0.001 respectively; unpaired t-test). For *Trichodorus* nematodes however, circularity did not significantly increase on exposure to PEITC (p = 0.052; unpaired t-test). The measurements indicate that circularity can be used as a parameter by which to measure toxin-induced decreases in activity of motile nematodes. Less active nematodes, such as *Trichodorus*, differentiate themselves from active species by circularity remaining near constant on exposure to chemical treatment*.* For this study we did not pursue the use of more complex shape descriptors such as tortuosity, or in-depth multidimensional trait analysis. The choice of traits studied however, demonstrates the potential of BSPIM analysis for the discrimination of feeding groups.

## Discussion

### Linking nematode behaviour to biospeckle patterns

In this study, biospeckle analyses have been used to describe several natural behavioural and morphological characteristics of nematodes. Firstly, nematodes that are not moving could be defined by the high circularity of their biospeckle pattern (illustrated in this paper by the inactive *Trichodorus* nematodes). As the size of living nematodes is directly reflected in the biospeckle pattern, this allows estimation of morphology and internal bioactivity to be assessed. Secondly, nematode motility could be detected as drawn-out biospeckle patterns with low circularity. This creates the possibility of measuring the length of these patterns and the nature of their trajectories in space. Furthermore, differences within the detected motility patterns can be observed for different nematode groups and how these patterns vary in time.

Bacterial feeder activity was shown to drop within 2 h after insertion into Ludox® TMA/water, while spontaneous movement was still being displayed after a week. Possibly, this decline in activity reflects a decline in exploratory activity over time. Bacterial feeders display scanning movements when perceiving stimulatory chemical cues in their environment [49] and it is likely that this behaviour declines once acclimatised. Changes in activity levels over 24 h were not seen for plant feeders and especially not for *Trichodorus* nematodes, whose low activity levels appeared to remain constant. *Trichodorus* nematodes clearly differentiated themselves by their lack of movement compared to samples of non-trichodorid mixed plant feeders or bacterial feeders, with biospeckle intensity remaining constant over time. This analysis confirmed behaviour observed under the light microscope in this and in previous studies [48, 59].

Our results also suggest that biospeckle can be used to measure changes in motility without direct tracking of bodies. First, it is possible to gauge spontaneous movement as biospeckle volume (measured here as the number of detected object voxels). Bioactivity resulting from movement (Fig. 3c) highlighted differences in behaviour between the tested groups. Toxins cause nematode movement to decrease, which is measurable as a drop in the biospeckle volume of the detected object. Immobility, as seen here as an effect of PEITC (Fig. 5aiii), can be deduced from the circularity of the biospeckle patterns. In the case of dead nematodes the biospeckle phenomenon ceases [47] and biospeckle intensity becomes negligible. Biospeckle can therefore be used to determine whether non-motile nematodes are still alive. Alternatively, nematodes internal bioactivity can be derived from Total Biospeckle Intensity (by summing the intensities of each detected object). Individually, the biospeckle intensity of detected nematodes (controls in Fig. 4d–f) showed less variability across groups and over time, possibly laying more emphasis on biospeckle produced by cellular processes. Intensity-based measures of bioactivity may therefore be more suitable for use in toxicology studies, while movement-based measures may better reflect species diversity.

### Target specificity and trophic level toxicity testing

Previous results show that biospeckle patterns can provide numerous independent and quantitative descriptors of the behaviour of nematodes. Unlike genomic analyses, these indicators can efficiently discriminate behaviours that more directly link to biological functions. Hence there is great potential to develop biospeckle approaches to analyse responses of nematode communities to stressors such as chemical treatments against plant pathogens.

In this study, we have tested the suitability of biospeckle. PEITC, a plant-derived nematicide, had a range of effects on nematode bioactivity. Despite being the target group, *Trichodorus* nematodes were the least susceptible to this chemical and less affected than the non-target bacterial feeder group. Application of 2-propenyl ITC in the field has previously been reported as not significantly affecting the abundance of *Trichodorus* populations [60] though it probably was deployed at much lower concentrations than in the present study. Plant feeders excluding trichodorid species were the most susceptible to PEITC, being the only group for which there was a significant effect after 2 h of treatment and showing a prolonged decline in biospeckle intensity. In the presence of methanol, *Trichodorus* nematodes again showed the highest tolerance amongst the tested groups. Methanol had a strong effect on both bacterial and non-trichodorid plant feeders. For *C. elegans*, a 12% methanol solution has been reported to be lethal to 50% of nematodes after 24 h [61]. This is in broad agreement with our finding that for bacterial feeders at around double that concentration only negligible biospeckle activity was observed after 24 h. Overall, bacterial feeder populations are reported to be more tolerant of pollution-induced stress than plant feeders [9]. This, however, appears to be largely due to plant feeders having lower reproductive capacity, while bacterial feeders tend to have high metabolic rates and the ability to re-populate contaminated soil quickly [61, 62]. In general, in the presence of stressors such as toxins, relative abundance is thought to shift in favour of colonising bacterial feeders [9]. Our study however suggests that susceptibility to toxins can vary significantly within trophic groups with *Trichodorus* nematodes showing greater resilience than either bacterial or non-trichodorid plant feeders.

The techniques developed here could be improved greatly by further automation, increase in diversity of light signals collected, and by the development of data science approaches to interpret the biospeckle patterns. Techniques such as microfluidics and cell sorting have seen major development recently [63, 64]. They have allowed automated and fast processing of cells and microscopic bodies and could be coupled to biospeckle data acquisition systems in the future. Also, there is potential to increase the numbers of independent indicators obtained from nematode activity, for example using multiple wavelengths, improved resolution or by combining other forms of live signals. Research in hyperspectral imaging for example, has demonstrated that the increase in the number of wavelengths in an imaging pipeline can significantly improve feature detection in image analysis [65, 66]. Such systems could feed large datasets of biospeckle patterns from nematodes extracted from soils. Finally, a database of biospeckle signals collected from a broad range of nematode species could be used to train sophisticated machine learning algorithms that efficiently read biospeckle signals, classify their patterns and associate them with functional groups. Similar approaches have been used, for example, to detect motile human parasites [67].

### Integrating bioactivity in soil health assessments

Soil health cannot be measured directly and frequent use has been made of biological indices such as composition of nematode communities [28, 68]. Significant spatial [69] and temporal variation, for example due to climatic conditions [70], and the lack of direct knowledge of their feeding habits [3], limits the use of univariate indices in the field [71]. Because current methods lack the ability to monitor live activity, several components with a net significant effect on soil function, remain underemphasized [72]. These include the interactions between organisms, their multi-functionality, the rates at which functions are carried out and how both function and rate vary with soil conditions.

There is potential, therefore, to develop diagnostic tools based on biospeckle principles to complement metagenomic approaches which lack the ability to measure a live response. Given that site conditions can change rapidly between sampling, current techniques that infer treatment effects by comparing relative nematode abundances, require a range of other variables, such as soil moisture and temperature to be considered [70, 73, 74]. By using live organisms and observing effects in real-time, however, BSPIM allows the bioactivity of different trophic or functional groups sampled in the field to be monitored under controlled laboratory conditions. This in turn allows predictions to be made on the optimal conditions, such as temperature or pH, for a given treatment before conducting a full-scale field trial. To develop the BSPIM technique into a tool for the holistic assessment of soil microorganism activity requires increasing its sensitivity to different forms of movement and behaviour with the aim of ascertaining nematodes’ trophic and functional groups. For example, more information is needed on the natural variability of nematode activity depending on soil temperature, moisture, and other seasonal changes in order to gauge the minimum effective dosage under current field conditions. More detailed differentiation of head movements from whole body movement on the other hand could give a better resolution of specific behaviours, particularly of feeding habits. BSPIM’s ability to distinguish internal from motility induced biospeckle activity also makes it very applicable to studies of dormancy. Here, however, the extraction of dormant nematodes from soil requires an alternative to the Baermann Funnel method used in this study. The technique would then offer greater insight into the variability of soil organism bioactivity and a quick, cost-effective development platform for targeted pest control.

## Conclusions

Biospeckle imaging has proved to be an efficient technique to detect changes in biological activity from a broad range of organisms without the need to know their specific genotypes. Here, we have expanded the technique to screen the efficacy of potential nematicides on soil-inhabiting nematodes with different feeding habits. Toxicological effects on nematode behaviour are revealed through changes in the biospeckle intensity, and the 3D patterns created by nematode motility. Biospeckle imaging is therefore a suitable candidate for broad, community-scale toxicity testing of in-soil organisms. Because measurement is carried out optically and is remote from the sample, it also lends itself to automation, thereby speeding up the drug discovery process. The method was also shown to be selective, with patterns detected for mixtures of plant and bacterial feeders suggesting biospeckle imaging could be used for automated analysis of natural activity patterns as well as their response to toxins.
